# A novel in vitro model for the assessment of postnatal myonuclear accretion

**DOI:** 10.1186/s13395-018-0151-4

**Published:** 2018-02-14

**Authors:** Anita Kneppers, Lex Verdijk, Chiel de Theije, Mark Corten, Ellis Gielen, Luc van Loon, Annemie Schols, Ramon Langen

**Affiliations:** 1 Department of Respiratory Medicine, NUTRIM School of Nutrition and Translational Research in Metabolism, Maastricht University Medical Centre+, Maastricht, The Netherlands; 2 Department of Human Biology and Movement Sciences, NUTRIM School of Nutrition and Translational Research in Metabolism, Maastricht University Medical Centre+, Maastricht, The Netherlands

**Keywords:** Skeletal muscle, Satellite cells, Cell differentiation, Cell fusion, Postnatal myogenesis, Muscle maintenance, Muscle regeneration, Muscle repair, Myotubes, Myoblasts

## Abstract

**Background:**

Due to the post-mitotic nature of myonuclei, postnatal myogenesis is essential for skeletal muscle growth, repair, and regeneration. This process is facilitated by satellite cells through proliferation, differentiation, and subsequent fusion with a pre-existing muscle fiber (i.e., myonuclear accretion). Current knowledge of myogenesis is primarily based on the in vitro formation of syncytia from myoblasts, which represents aspects of developmental myogenesis, but may incompletely portray postnatal myogenesis. Therefore, we aimed to develop an in vitro model that better reflects postnatal myogenesis, to study the cell intrinsic and extrinsic processes and signaling involved in the regulation of postnatal myogenesis.

**Methods:**

Proliferating C2C12 myoblasts were trypsinized and co-cultured for 3 days with 5 days differentiated C2C12 myotubes. Postnatal myonuclear accretion was visually assessed by live cell time-lapse imaging and cell tracing by cell labeling with Vybrant® DiD and DiO. Furthermore, a Cre/LoxP-based cell system was developed to semi-quantitatively assess in vitro postnatal myonuclear accretion by the conditional expression of luciferase upon myoblast–myotube fusion. Luciferase activity was assessed luminometrically and corrected for total protein content.

**Results:**

Live cell time-lapse imaging, staining-based cell tracing, and recombination-dependent luciferase activity, showed the occurrence of postnatal myonuclear accretion in vitro. Treatment of co-cultures with the myogenic factor IGF-I (*p* < 0.001) and the cytokines IL-13 (*p* < 0.05) and IL-4 (*p* < 0.001) increased postnatal myonuclear accretion, while the myogenic inhibitors cytochalasin D (*p* < 0.001), myostatin (*p* < 0.05), and TNFα (*p* < 0.001) decreased postnatal myonuclear accretion. Furthermore, postnatal myonuclear accretion was increased upon recovery from electrical pulse stimulation-induced fiber damage (*p* < 0.001) and LY29004-induced atrophy (*p* < 0.001). Moreover, cell type-specific siRNA-mediated knockdown of myomaker in myoblasts (*p* < 0.001), but not in myotubes, decreased postnatal myonuclear accretion.

**Conclusions:**

We developed a physiologically relevant, sensitive, high-throughput cell system for semi-quantitative assessment of in vitro postnatal myonuclear accretion, which can be used to mimic physiological myogenesis triggers, and can distinguish the cell type-specific roles of signals and responses in the regulation of postnatal myogenesis. As such, this method is suitable for both basal and translational research on the regulation of postnatal myogenesis, and will improve our understanding of muscle pathologies that result from impaired satellite cell number or function.

**Electronic supplementary material:**

The online version of this article (10.1186/s13395-018-0151-4) contains supplementary material, which is available to authorized users.

## Background

Skeletal muscle fibers arise from the biochemical and morphological differentiation of muscle precursor cells. This process is called myogenesis and is essential for prenatal development, as well as postnatal muscle tissue growth, maintenance, and repair.

Myogenesis during prenatal skeletal muscle development is elaborately studied (for review, see, e.g., Buckingham et al. [1]). Briefly, limb muscles arise from muscle progenitor cells that are derived from the dermomyotome—the maturing somite that forms from the paraxial mesoderm. Upon external cues, muscle progenitor cells migrate into the limb bud, proliferate, become committed to the myogenic transcription program, differentiate biochemically and morphologically, and fuse with each other to give rise to primary and secondary muscle fibers.

In postnatal skeletal muscle, muscle progenitor cells are also present. These adult progenitor cells, called satellite cells, originate from the same dermomyotome-derived Pax3+/Pax7+ population [2–5] and are located between the sarcolemma and basal lamina. In the early postnatal period, myogenesis facilitates muscle growth by satellite cell proliferation, differentiation, and fusion with a pre-existing muscle fiber [6], which is recapitulated during adult muscle hypertrophy. However, in healthy adult muscle, the majority of satellite cells are quiescent. Upon external cues, adult satellite cells become activated, proliferate, and either return to quiescence in a process called self-renewal, or differentiate to fuse with a pre-existing muscle fiber (for review, see, e.g., Dhawan et al. [7]).

Due to the post-mitotic nature of myonuclei, postnatal myogenesis is essential for skeletal muscle maintenance, growth, repair, and regeneration. In vivo in human skeletal muscle, these roles are demonstrated by the acute increase in satellite cell activation status and number after exercise, and the positive association between changes in muscle fiber size, satellite cell content, and myonuclear content after prolonged exercise training, as reviewed by Snijders et al. [8]. The requirement of satellite cells for muscle growth, repair, and regeneration has mainly been established in animal studies. These fundamental studies showed that satellite cell ablation by γ-irradiation or Pax7+ cell depletion prevents sustained overload-induced muscle hypertrophy and recovery from muscle injury [9–12]. Although subsequent work has challenged this concept [13], more recent studies confirm the essential role of satellite cells in muscle growth [14, 15].

A role for impaired myogenesis, by either a reduced satellite cell number or function, has been implicated in the loss of skeletal muscle mass and function (i.e., sarcopenia) in aging, chronic diseases, and myopathies [16–18]. The identification of regulators and pathways involved in postnatal satellite cell function and dysfunction is imperative for a better comprehension of the role of myogenesis in aging and disease-related muscle loss, and to develop targeted intervention strategies aimed at optimizing muscle health and function.

Considerable progress has been made towards uncovering these mediators and regulators of satellite cell function in health and disease, as reviewed by Buckingham et al. [1]. In vivo and ex vivo models were fundamental in their identification, while knowledge on the causal role of these mediators and regulators in myogenesis was primarily based on in vitro culturing and differentiation of primary satellite cells and immortalized myoblasts. This in vitro formation of syncytia from mononucleated myoblasts morphologically resembles developmental myogenesis, but has also been employed as a model to study the regulation of postnatal myogenesis [19–21]. Indeed, it was theorized that muscle regeneration recapitulates developmental myogenesis. However, based on the reported differential requirements for β-catenin during embryonic and fetal myogenesis [5], this theory has been challenged [22–24]. Several reviews compared developmental and postnatal myogenesis and found important distinctions in the involved cells, anatomy, cellular mechanisms, and expression of regulatory genes [22, 24, 25]. This novel insight requires reframing of the current knowledge on myogenesis, and reconsideration of established myogenesis models. Specifically, we should consider the contextual use of the classical in vitro model of myogenesis, i.e., syncytia formation from myoblasts, which may represent developmental myogenesis, but incompletely or even inaccurately portrays postnatal myogenesis.

The use of a more appropriate model will advance the comprehension of regulation and dysregulation of postnatal myogenesis and its role in the development of sarcopenia, and is thus indispensable in the identification of specific targets for effective prevention and treatment of sarcopenia. In this study, we therefore aimed to develop an in vitro model that better reflects postnatal myogenesis, to study the cell intrinsic and extrinsic processes and signaling involved in the regulation of postnatal myogenesis.

## Methods

### Cell culture and reagents

Cell maintenance and experiments were performed in a humidified incubator at 37 °C with 5% CO_2_. C2C12 myoblasts (ATCC, Wesel, Germany; #CRL-1772) were maintained in growth medium (GM) which was composed of Dulbecco’s modified Eagle’s medium (DMEM 1 g/L glucose (Gibco, Rockville, MD; #22320-022) supplemented with 9% (*v*/*v*) fetal bovine serum (FBS), and 50 U/mL penicillin and 50 μg/mL streptomycin (P/S) (Gibco). To induce differentiation, cells were plated onto tissue culture plates coated with 2% (*v*/*v*) Growth Factor Reduced Matrigel® (BD Biosciences, Bedford, MA) and switched to differentiation medium (DM) composed of DMEM 4.5 g/L glucose (Gibco; #41966-029) supplemented with 0.5% (*v*/*v*) heat-inactivated FBS and P/S.

Human C25 myoblasts (HSM) [26] were kindly provided by V. Mouly. HSM cells were maintained in GM which was composed of Skeletal Muscle Cell Growth Medium supplemented with 42 μg/mL bovine Fetuin, 8.3 ng/mL human recombinant Epidermal Growth Factor, 0.83 ng/mL human recombinant Basic Fibroblast Growth Factor, 8.3 μg/mL human recombinant Insulin, 0.33 μg/mL Dexamethasone (all from PromoCell, Heidelberg, Germany), 17% (*v*/*v*) FBS, and P/S. To induce differentiation, cells were plated onto Matrigel-coated tissue culture plates and switched to DM which was composed of DMEM 4.5 g/L glucose, GlutaMAX™ (Gibco; #61965-026) supplemented with P/S.

DM was replaced 24 h after initiation of differentiation, and subsequently every 48 h. Myotube–myoblast co-cultures were initiated with 5 days differentiated myotubes and 5000 cells/cm^2^ myoblasts, unless indicated otherwise.

For lentivirus production, 293FT cells (Invitrogen, Carlsbad, CA; #R700-07) were maintained in DMEM 4.5 g/L glucose supplemented with 9% (*v*/v) FBS, P/S, and 500 μg/mL Geneticin (Gibco).

Recombinant human insulin-like growth factor 1 (IGF-I) (Sigma-Aldrich, Zwijndrecht, The Netherlands), recombinant human/mouse/rat growth differentiation factor 8 (Myostatin, MSTN), recombinant mouse interleukin-4 (IL-4) and human IL-13 (R&D systems, Minneapolis, MN), and recombinant mouse tumor necrosis factor alpha (TNF-α) (Merck Millipore, Amsterdam, The Netherlands) were dissolved in 0.1% bovine serum albumin (BSA) (Sigma-Aldrich) in Hanks’ Balanced Salt Solution (HBSS). LY294002 (LY) (Merck Millipore) and Cytochalasin D (CytoD) (Sigma-Aldrich) were dissolved in DMSO.

### Electrical pulse stimulation

Myotubes were electrically stimulated using a C-Pace unit and a C-Dish electrode assembly for 35 mm culture dishes (Ion Optix, Milton, MA). Stimulation was performed according to the “twitch” protocol by Orfanos et al. [27]. Briefly, pulses were applied for 20 ms at 10 V, at a frequency of 1 Hz.

### RNA interference

Knockdown of Myomaker was achieved by RNA interference. Target Silencer® Select siRNA or negative control Silencer® Select siRNA (final 10 nM) was mixed with Lipofectamine RNAiMAX (Invitrogen) in Opti-MEM reduced serum medium (Gibco; #31985-070). After complex formation at room temperature (RT) for 5 min, the transfection mix was added to adherent cells, and incubated for 24 h before initiation of co-culturing.

### Western blot

Cells were lysed in whole cell lysis buffer (20 mM Tris, 150 mM NaCl, 1% Nonidet P40, and protease and phosphatase inhibitors (Roche)), incubated on ice for 30 min, and centrifuged at 14,000*g* for 30 min. Total protein concentration in the supernatant was determined using BCA Protein Assay kit (Pierce) according to the manufacturer’s instructions. 4× Laemmli sample buffer (0.25 M Tris-HCL ph 6.8, 8% (*w*/*v*) SDS, 40% (*v*/*v*) glycerol, 0.4 M DTT, and 0.02% (*w*/*v*) Bromophenol Blue) was added to the supernatant, and samples were heated to 100 °C for 5 min. Protein (3 μg myoblast lysate; 10 μg myotube lysate) were separated on a Criterion XT Precast 4–12% Bis-Tris gel (Bio-Rad), followed by transfer to a 0.45 μm nitrocellulose membrane (Bio-Rad) by electroblotting. For total protein detection, the membrane was stained with PonceauS solution (0.2% PonceauS in 1% acetic acid; Sigma-Aldrich Chemie) and imaged using the Amersham imager 600RGB. The membrane was blocked for 1 h at RT in Tris-buffered saline with Tween20 (20 mM Tris, 137 mM NaCl, 0.1% (*v*/*v*) Tween20, pH 7.6 (TBST)) containing 5% (*w*/*v*) nonfat dry milk (Campina). After washing in TBST, the membrane was incubated overnight at 4 °C with 1:1000 anti-Myomaker (NBP2-34175; Novus) diluted in TBST with 5% (*w*/*v*) BSA. Subsequently, the membrane was incubated with peroxidase conjugated secondary antibody solution (PI-1000; Vector laboratories) for 1 h at RT, and the target was visualized by chemiluminescence using supersignal FEMTO chemiluminescent substrate (Pierce Biotechnology, Inc.) according to the manufacturer’s instructions, and detected using the Amersham Imager 600RGB. Signals were quantified with Image Quant Software (Amersham), and corrected for total protein content.

### Stable transfection

The LV-floxed-Luc C2C12 cell line expresses a conditional luciferase cassette dependent on Cre-mediated recombination, and was generated by Lipofectamine® 2000 (Invitrogen) mediated transfection with LV-floxed-Luc (Addgene, Cambridge, MA; #60622) and subsequent selection based on a selectable marker.

The Cre C2C12 cell line expresses Cre recombinase, and was obtained by lentivirus-mediated infection with Cre-IRES-PuroR (Addgene; #30205). Lentiviral supernatant was produced by polyethylenimine-mediated co-transfection of 293FT cells with Cre-IRES-PuroR, pRSV-Rev (Addgene; #12253), pMDLg/pRRE (Addgene; #12251), and pMD2.G (Addgene; #12259) (1:1:1:1) in C2C12 GM. Lentivirus supernatant was collected 24, 48, and 72 h after transfection, filter sterilized (0.45 μM), and stored at − 20 °C. C2C12 cells were infected by addition of lentiviral supernatant in GM (1:1) containing 1 μg/mL polybrene (Sigma-Aldrich), and subsequently selected based on a selectable marker.

Stable polyclonal cell lines were expanded, and stored in liquid nitrogen. For each experiment, a new vial was used to reduce variability between experiments.

### Cell staining and hybrid quantification

C2C12 or HSM cells were subjected to live staining with Vybrant® DiD or DiO cell labeling solution (Life Technologies, Carlsbad, CA) before plating. Briefly, myoblasts were stained in suspension in GM at 37 °C for 20 min, and washed three times by pelleting and resuspension in GM. To obtain stained myotubes, stained myoblasts were plated and differentiated as described. Subsequently, co-culturing was initiated, and after 2 days, cell monolayers were washed with HBSS and fixed in 3.7% paraformaldehyde (PFA). After fixation, nuclei were stained with DAPI and coverslips were mounted onto the cell monolayers using Mowiol/DABCO.

Images were taken at a 100× magnification using an Eclipse E800 microscope (Nikon) connected to a digital camera (DXM, 1200 NF, Nikon). For quantification of hybrid formation, five random fields of view (FOV) were captured from each well. Myotubes (i.e., cells with ≥ 3 nuclei) containing both DiD and DiO cell tracers, resulting from myonuclear accretion, were defined as hybrids. Total myotubes and hybrid myotubes were counted, and hybrids were expressed as percentage of the total number of myotubes. The sum of five images was used to represent each well.

### BrdU labeling and immunohistochemical detection

C2C12 myoblasts were incubated with 10 μM bromodeoxyuridine (BrdU) (Sigma-Aldrich) in GM for 24 h, and subsequently co-cultured with 5 days differentiated myotubes.

At the indicated time points, cells were fixed in 3.7% PFA, and subsequently permeabilized with 0.1% Triton X-100 (Sigma-Aldrich) in phosphate buffered saline (PBS). Fixed cells were denatured with 1 N HCL in PBS for 30 min at 45 °C, and neutralized with 0.1 M borate buffer (pH 8.5) for 10 min at RT. After 1 h incubation with blocking buffer (1% BSA, 22.52 mg/ml glycine, 0.1% Tween in PBS (PBST)), cells were incubated overnight with rat monoclonal anti-BrdU antibody (Abcam; #ab6326) (1:75 in 1% BSA in PBS) at 4 °C in a humidified chamber, and then incubated for 1 h with rabbit anti-rat antibody (Abcam; #ab6730) (1:200 in 1% BSA in PBS) at RT in the dark. Nuclei were subsequently stained with DAPI, and coverslips were mounted onto the cell monolayers using Mowiol/DABCO. Images were taken at a 100× magnification using an Axio Observer A1 microscope (Zeiss) connected to a digital camera (AxioCam ICM1, Zeiss). From each well, five random FOV were captured, and BrdU+ nuclei (staining positive for BrdU and DAPI) in myotubes (i.e., cells with ≥ 3 nuclei) were counted. The sum of five images was used to represent each well.

### Luciferase detection

Cell monolayers of Cre and LV-floxed-Lux C2C12 co-cultures were lysed in reporter lysis buffer (Promega, Madison, WI) by scraping and a subsequent freeze-thaw cycle, and briefly centrifuged to remove cell debris. Luciferase activity was measured by addition of 100 μL luciferase assay reagent (1.07 mM MgCO_3_, 2.67 mM MgSO_4_, 20.0 mM Tricin, 0.10 mM EDTA, 33.3 mM DTT, 530 μM ATP, 270 μM Coenzyme A, 470 μM Luciferin) to 20 μL lysate, and subsequent luminescence detection using a single tube luminometer (Lumat LB 9507, Berthold, Bad Wildbad, Germany), according to the manufacturer’s protocol (Promega). The luminescence signal (RLU) was corrected for total protein content in the soluble fraction assessed by a BCA protein assay (Pierce Biotechnology, Rockford, IL).

### Statistics

Data are presented as means ± SEM and are representative of ≥ 3 independent experiments. Analyses were performed using SPSS Statistics (version 22.0, IBM Corp., Armonk, NY). Comparisons between two groups were performed using an independent student’s t-test. Comparisons between > 2 groups were performed by one-way ANOVA with Bonferroni post hoc correction. A *p* value < 0.05 was considered statistically significant.

## Results

### In vitro fusion of myoblasts with myotubes

**Fig. 1:**
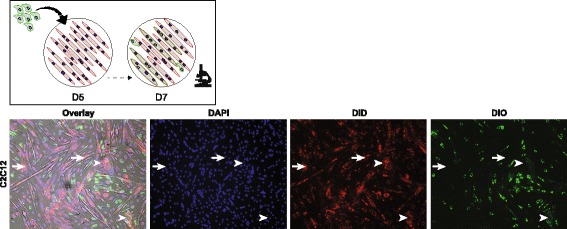
In vitro myoblast–myotube fusion. Hybrid formation in DiD-stained C2C12 myotubes 2 days after initiation of co-culturing with DiO-stained C2C12 myoblasts. (DAPI/nuclei: blue; DiD: red; DiO: green). Arrows indicate non-hybrid myotubes, arrow heads indicate hybrid myotubes

The classical in vitro myogenesis model entails the formation of syncytia from myoblasts. To better mimic postnatal myogenesis in vitro, we sought to represent the involved fusion partners. To this end, myotubes obtained by 5-day differentiation of C2C12 myoblasts were co-cultured with yet undifferentiated myoblasts. Through live cell time-lapse, imaging fusion of myoblasts with myotubes was observed during the 48 h after initiation of co-culturing (Additional file 1; Additional file 2: Figure S1). Accordingly, the fusion of DiO-stained C2C12 myoblasts with DiD-stained myotubes resulted in the formation of hybrid myotubes (Fig. 1), and in vitro myotube–myoblast fusion was confirmed in a similar experiment in HSM cells (Additional file 3: Figure S2). Together, this shows that both C2C12 and HSM cells are capable of in vitro postnatal myonuclear accretion. (Additional file 1).

### In vitro postnatal myonuclear accretion is increased by IGF-I

**Fig. 2:**
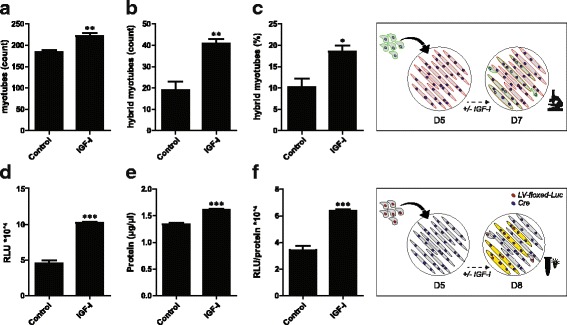
Increased in vitro postnatal myonuclear accretion in C2C12 cells upon IGF-I treatment. **a-c** Staining-based assessment of myonuclear accretion 2 days after initiation of co-culturing +/- 10 nM IGF-I. **a** total number of myotubes, **b** number of hybrid myotubes, **c** % hybrid myotubes. **d-f** Luciferase-based assessment of myonuclear accretion 3 days after initiation of co-culturing +/− 10 nM IGF-I. D) luciferase activity (RLU) per well, E) protein content (μg/μL) per well, F) relative luciferase activity (RLU/protein content) per well. Values are means ± SEM, *n* = 4. *p < 0.05, **p < 0.01, ***p < 0.001

Staining-based quantification was optimized (Additional file 4: Figure S3), and used to assess if the number of in vitro postnatal myonuclear accretion events can be modified. Co-cultures were treated with IGF-I, representing a well-established myogenic factor, which impacts on both proliferation and differentiation [28]. This revealed a higher total amount of myotubes, a higher total amount of hybrids, and a higher relative amount of hybrids 2 days after initiation of co-culturing in the presence of IGF-I (Fig. 2a–c). IGF-I treatment started 24 h after initiation of co-culturing had no effect, whereas 24-h pre-treatment with IGF-I increased the number of myotubes but did not affect the relative amount of hybrid myotubes (Additional file 5: Figure S4). This showed that the staining-based method had sufficient power to detect relevant differences in postnatal myonuclear accretion. Furthermore, the staining-based method displayed a significant inter-rater correlation and a moderate to high inter-rater agreement (Additional file 6: Figure S5). However, Bland-Altman analysis revealed a significant fixed bias for both the absolute and relative amount of hybrids, and potentially clinically relevant differences may lie within the 95% limits of agreement (Additional file 6: Figure S5D, F). Moreover, the staining-based assessment of postnatal myonuclear accretion was labor intensive and time consuming. For unbiased, high throughput, semi-quantitative assessment of postnatal myonuclear accretion, we therefore developed a Cre/LoxP-based cell fusion reporter system (Additional file 7: Figure S6), which allows the conditional expression of luciferase after myoblast–myotube fusion. IGF-I treatment of LV-floxed-Luc myotubes and Cre myoblast co-cultures increased protein content and absolute luciferase activity, but no change in the relative luciferase activity was observed. However, IGF-I treatment of Cre myotube and LV-floxed-Luc myoblast co-cultures resulted in an increased protein content, and increased relative and absolute luciferase activity in cells lysed 3 days after initiation of co-culturing (Fig. 2d–f, Additional file 7: Figure S6F−H), indicating increased cell fusion.

The Cre/LoxP-based cells system was further characterized by employing co-cultures with increasing concentrations of IGF-I. A dose-dependent increase in the relative luciferase activity and total protein content was observed upon treatment with increasing concentrations of IGF-I (Additional file 8: Figure S7 d−f). To verify IGF-I stimulated myonuclear accretion, the incorporation of BrdU-labeled myoblast nuclei into myotubes was assessed. A dose-dependent increase in the number of BrdU+ nuclei in myotubes was observed upon treatment with IGF-I, which had a strong, but non-significant correlation with the change in relative luciferase activity (Additional file 8: Figure S7 G−I). To further validate luciferase activity in the Cre/LoxP-based model as an index of fusion, the stimulatory effects of IGF-I on protein synthesis- and fusion-dependent luciferase expression were dissected. Cre and LV-floxed-Luc myoblasts were co-cultured and differentiated into hybrid myotubes with constitutive luciferase expression, and their subsequent time- and dose-dependent luciferase activity in response to IGF-I was compared to myoblast-myotube co-cultures (Additional file 9: Figure S8). Although IGF-I treatment increased the relative luciferase activity at *T* = 72 in both systems, the magnitude of the effect in the preformed hybrid myotubes was negligible compared to that observed in fusion-dependent luciferase expressing co-cultures (Additional file 9: Figure S8).

### In vitro postnatal myonuclear accretion is reduced by cytochalasin D, myostatin and TNFα

**Fig. 3:**
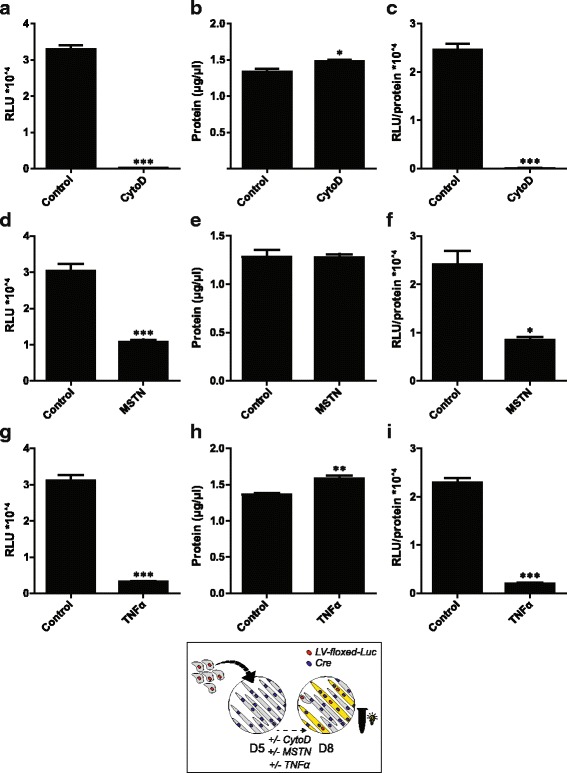
Decreased in vitro postnatal myonuclear accretion in C2C12 cells upon CytoD, MSTN and TNFα treatment. Luciferase-based assessment of myonuclear accretion 3 days after initiation of co-culturing +/- 0.3 μM CytoD (A-C), 250 ng/mL MSTN (D-F), or 10 ng/mL TNFα (G-I). **a** Luciferase activity (RLU) per well, **b** protein content (μg/μL) per well, **c** relative luciferase activity (RLU/protein content) per well, **d** luciferase activity (RLU) per well, **e** protein content (μg/μL) per well, **f** relative luciferase activity (RLU/protein content) per well, **g** luciferase activity (RLU) per well, **h** protein content (μg/μL) per well, **i** relative luciferase activity (RLU/protein content) per well. Values are means ± SEM, *n* = 4. *p < 0.05, **p < 0.01, ***p < 0.001

To assess if the rate of in vitro postnatal myonuclear accretion can be suppressed, co-cultures were treated with known inhibitors of myogenesis: CytoD, MSTN and TNFα. CytoD treatment upon initiation of co-culturing resulted in a reduction of luciferase activity to background levels measured in myotube only cultures, and a small increase in total protein content (Fig. 3a–c). MSTN treatment upon initiation of co-culturing had no effect on the protein content, but led to a decrease in absolute and relative fusion reporter activity (Fig. 3d–f). Similarly, TNFα treatment upon initiation of co-culturing led to a decrease in absolute and relative fusion reporter activity, in presence of an increase in the total protein content per well (Fig. 3g–i). Suppression of fusion reporter activity by TNFα treatment was dose-dependent (Additional file 8: Figure S7 A−C). Moreover, when applied to preformed hybrid myotube cultures no decrease in absolute or negative luciferase content was detected in response to TNFα (Additional file 9: Figure S8 D−F), suggesting reduced fusion-dependent luciferase expression in myoblast-myotube co-cultures (Fig. 3g−i) rather than decreased synthesis of luciferase protein.

### In vitro postnatal myonuclear accretion is increased by the anti-inflammatory cytokines IL-13 and IL-4

**Fig. 4:**
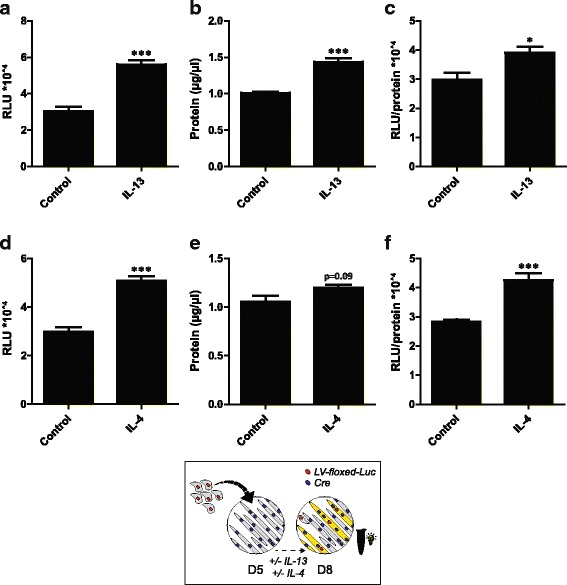
Increased in vitro postnatal myonuclear accretion in C2C12 cells upon IL-13 and IL-4 treatment. Luciferase-based assessment of myonuclear accretion 3 days after initiation of co-culturing +/− 250 ng/mL IL-13 (**a-c**) or 500 ng/mL IL-4 (**d-f**). **a** Luciferase activity (RLU) per well, **b** protein content (μg/μL) per well, **c** relative luciferase activity (RLU/protein content) per well, **d** luciferase activity (RLU) per well, **e** protein content (μg/μL) per well, **f** relative luciferase activity (RLU/protein content) per well. Values are means ± SEM, *n* = 4. *p < 0.05, ***p < 0.001

In contrast to the pro-inflammatory cytokine TNFα, the anti-inflammatory cytokines IL-13 and IL-4 have been identified as promoting factors for postnatal myogenesis [29, 30]. IL-13 treatment upon initiation of co-culturing resulted in an increase in the total protein content and an increase in the absolute and relative fusion reporter activity (Fig. 4a–c). Similarly, IL-4 treatment tended to increase the total protein content and led to an increase in the absolute and relative fusion reporter activity (Fig. 4d–f).

### In vitro postnatal myonuclear accretion is increased upon recovery from myotube injury and atrophy

**Fig. 5:**
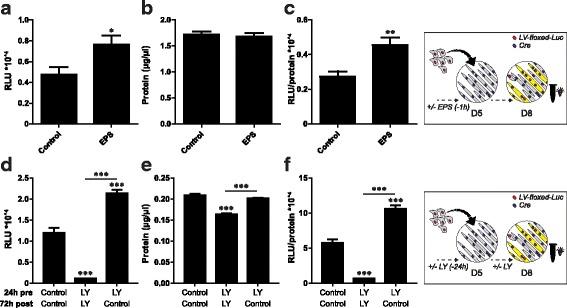
Increased in vitro postnatal myonuclear accretion in C2C12 cells upon recovery from myotube damage and atrophy. Luciferase-based assessment of myonuclear accretion 3 days after initiation of co-culturing of myotubes pre-treated by 1-h electrical pulse stimulation, with myoblasts in presence of conditioned medium (*n* = 6). **a** Luciferase activity (RLU) per well, **b** protein content (μg/μL) per well, **c** relative luciferase activity (RLU/protein content) per well. Luciferase-based assessment of myonuclear accretion 3 days after initiation of co-culturing of myotubes pre-treated +/- 15 μM LY (24 h pre), with myoblasts in presence or absence of 15 μM LY (72 h post) (n = 4). **d** luciferase activity (RLU) per well, **e** protein content (μg/μL) per well, **f** relative luciferase activity (RLU/protein content) per well. T = initiation and duration of treatment (h) relative to start of co-culturing. Values are means ± SEM, *n* = 4. *p < 0.05, **p < 0.01, ***p < 0.001 compared to control or between indicated groups

Physiological conditions that trigger in vivo postnatal myonuclear accretion include the recovery from muscle damaging exercise such as eccentric exercise and the recovery from muscle atrophy. In vitro myotube damage induced by electrical pulse stimulation (EPS) followed by recovery in the presence of myoblasts led to an increased total protein content and increased absolute and relative fusion reporter activity compared to untreated co-cultures (Fig. 5a–c). In vitro induction of atrophy by LY pre-treatment and maintenance during co-culturing led to a significant decrease in the total protein content and a decrease in the absolute and relative fusion reporter activity (Fig. 5d–f). Furthermore, removal of LY upon initiation of co-culturing led to a normalization of the total protein content and an increase in the absolute and relative fusion reporter activity compared to maintained LY stimulation and control (Fig. 5d–f).

### In vitro postnatal myonuclear accretion requires the expression of Myomaker in myoblasts

**Fig. 6:**
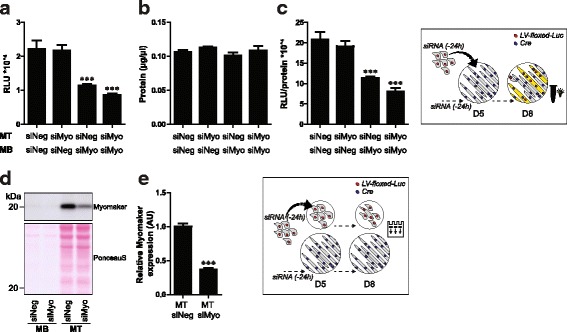
Myomaker is specifically required in myoblasts for in vitro postnatal myonuclear accretion in C2C12 cells. Luciferase-based assessment of myonuclear accretion 3 days after initiation of co-culturing of myotubes (MT) with myoblasts (MB) which received 24-h pre-treatment with either negative control siRNA (siNeg) or siRNA against Myomaker (siMyo) (*n* = 4). **a** Luciferase activity (RLU) per well, **b** protein content (μg/μL) per well, **c** relative luciferase activity (RLU/protein content) per well. Parallel experiment of siRNA treated MT and MB, maintained in separate cultures. **d** Western blot of Myomaker, **e** densitometric analysis of 20 kDa band (*n* = 8). Values are means ± SEM. ***p < 0.001 compared to control

A novel factor involved in myogenic fusion that is required for muscle regeneration and hypertrophy is Myomaker [15, 31]. A cell type-specific differential requirement of Myomaker has been reported [32]. We assessed if the requirement of Myomaker for fusion is myoblast or myotube specific. SiRNA-mediated knockdown of Myomaker was verified and resulted in a strong reduction in Myomaker protein abundance in myotubes, whereas Myomaker expression was below the detection limit in myoblasts (Fig. 6d, e). Knockdown of Myomaker in myotubes did not alter the absolute and relative cell fusion reporter activity in co-cultures (Fig. 6a−c). In contrast, treatment of myoblasts with siRNA against Myomaker decreased both the absolute and relative fusion reporter activity in co-cultures containing Myomaker-expressing or -silenced myotubes (Fig. 6a−c). These data indicate that Myomaker expression in myoblasts is required for cell fusion.

## Discussion

The requirement for postnatal myogenesis in muscle growth, repair and regeneration, and basal muscle maintenance is evident from research in humans [8, 33, 34] and rodents [9–12, 35]. However, data on the regulation and dysregulation of postnatal myonuclear accretion is scarce due to the technical challenge to investigate and quantitate this process in vivo and in vitro. Although, myoblast–myotube fusion is frequently mentioned in literature, the majority of these in vitro studies actually refer to late myogenesis in progressively differentiating cultures. Certainly, these classical studies have provided invaluable insights in the regulation and dysregulation of myogenesis, however, their translatability to adult skeletal muscle maintenance can be challenged [5, 22–24].

In the current study, we developed an unbiased, high throughput, Cre/LoxP-based in vitro model system for semi-quantitative assessment of postnatal myonuclear accretion. This model builds on the broadly employed classical model of myogenesis, by including the fusion partners involved in in vivo postnatal myonuclear accretion through co-culturing of differentiated C2C12 myotubes with yet undifferentiated C2C12 myoblasts. Via live cell time-lapse imaging, staining-based cell tracing, and recombination-dependent luciferase activity, we show that postnatal myonuclear accretion occurs in vitro. This finding is in line with the scant previous studies assessing myoblast–myotube fusion in co-cultures by staining-based cell tracing [30, 36–39]. Furthermore, we reproduced this finding in a HSM cell line, and thereby confirm that in vitro myoblast–myotube fusion is not a C2C12 cell line specific anomaly.

Using this co-culture model, a substantial number of hybrid cells and a pronounced luciferase signal are detected even in the absence of an additional myogenic trigger. Although previous literature states that in uninjured adult muscles, satellite cells are mitotically quiescent [12], in line with our data, a more recent study challenged this proposed low basal myonuclear turnover rate and shows that a significant percentage of satellite cells divide and fuse in uninjured adult muscles [40]. Differences in observed “basal” myonuclear turnover rates may arise from variations in the employed methodologies. Importantly, satellite cells were reported to rapidly activate in explant cultures [41], which resembles the proliferative state of myoblasts in our co-culture model.

Based on the basal luciferase signal and the responsiveness of the Cre/LoxP-based reporter system with Cre and LV-floxed-Luc C2C12 alternating as acceptor and donor cells for myonuclear accretion, we conclude that co-culturing of Cre myotubes with LV-floxed-Luc myoblasts most sensitively reports fusion (Additional file 7: Figure S6). This likely reflects the recombination-dependent increase in luciferase expression in Cre myotubes with the incorporation of every additional nucleus derived from an LV-floxed-Luc myoblast, whereas accretion of one single nucleus derived from a Cre myoblast may result in the recombination of multiple nuclei present in one LV-floxed-Luc myotube. We show that in this model, IGF-I induces a profound increase in postnatal myonuclear accretion. IGF-I is a well-established driver of muscle hypertrophy that has been associated with increases in both protein synthesis and myogenesis [42]. In line with this, IGF-I treatment during co-culturing increases the total protein content, as well as cell fusion assessed by staining-based and reporter-based fusion quantification. In agreement with previous literature [42], the effect of IGF-I depends on the timing of treatment initiation, which may suggest a role for temporal IGF-I expression during postnatal myogenesis. Furthermore, we show that TNFα treatment decreases postnatal myonuclear accretion. This disease-related factor was previously shown to reduce the myogenic index [19]. Although we demonstrate a dose-dependent decrease in fusion upon increasing TNFα concentrations, a bimodal effect of TNFα treatment was found when lower concentrations were included [43]. Furthermore, positive effects of TNFα on proliferation have been reported [44], which may explain the increase in total protein content in TNFα-treated co-cultures.

These modulatory effects of IGF-I and TNFα show that the Cre/LoxP-based fusion reporter system is responsive to physiologically relevant ligands that modulate myogenesis. Furthermore, the IGF-I and TNFα-dose dependent (Additional file 8: Figure S7 A−F), and the myoblast-density dependent (Additional file 7: Figure S6 E) effects on the relative luciferase activity, together with the lag time for effects of modulations to result in changes in luciferase activity (Additional file 7: Figure S6 F−H, Additional file 9: Figure S8 A−C), suggest that the relative luciferase activity is reflecting fusion. Moreover, in keeping with previous research [32, 45], treatment of co-cultures with CytoD reduces the luciferase reporter activity to background levels, verifying that luciferase reporter activity in our system requires cell fusion. In line, the stimulation of luciferase activity corresponds with an increased presence of myoblast-derived myonuclei in myotubes, providing further support that alterations in luciferase activity are determined by, and therefore reflect fusion. Nevertheless, the absolute luciferase activity is a product of post-recombinational luciferase expression, which may be affected by alterations in cellular transcriptional and translational activity. To disentangle effects on fusion and protein synthesis, we compared the time-dependent effects of IGF-I and TNFα on fusion-dependent luciferase expressing co-cultures, and Cre and LV-floxed-Luc hybrid myotubes with constitutive luciferase expression, respectively (Additional file 9: Figure S8). Based on the large difference in the magnitude of the effect of IGF-I, and the difference in the direction of the effect of TNFα on the relative luciferase activity, we conclude that changes in the relative luciferase activity in the Cre/LoxP-based fusion reporter system are indeed mainly dictated by changes in fusion. Nevertheless, when the absolute rate of myonuclear accretion is of interest, luciferase-based screening should be followed up by quantitative assessment of myonuclear accretion, e.g., by staining-based nuclear tracing of BrdU labeled myoblasts.

The current method reports the cumulative functional contribution of fusion events, and may thereby facilitate the elucidation of the homeostatic control of myonuclear number, which is of particular interest for the slow progressive muscle wasting seen in aging [8], chronic diseases [46], and muscle dystrophy [47]. However, luciferase-based reporting of fusion operates with a certain lag time, determined by recombination, transcription, and translation of the reporter gene. Therefore, no complete distinction can be made between alterations in the amount or rate of myonuclear accretion, although both may be equally relevant. To gain a more mechanistic insight in the regulation of postnatal myonuclear accretion, the current method should be used in conjunction with assays for proliferation and biochemical differentiation, to distinguish between effects on those processes and fusion, as alterations in any of these processes will affect myonuclear accretion. Furthermore, it should be taken into account that long-term monitoring of in vitro myonuclear accretion is restricted by the limited cell viability in culture. Moreover, in contrast to the staining-based assessment of in vitro postnatal myonuclear accretion, Cre/LoxP-based assessment does not provide any insight in the intermediate morphological states of myoblasts before fusion with a myotube. Thus, although we refer to myoblast-myotube fusion, we cannot exclude the occurrence nor assess the relevance of, e.g., myoblast-myoblast fusion before fusion with a pre-existing myotube. Nevertheless, a major advantage of the Cre/LoxP-based system is that it does not rely on visual identification of individual myotubes which is labor intensive and complex due to irregular myotube structure, and it may therefore also be suitable to study postnatal myonuclear accretion in, e.g., 3D culture systems [48].

In an initial utilization of the model, we assessed pharmacological and physiological modulators of postnatal myonuclear accretion. Similar to IGF-I [49, 50], MSTN modulation is part of the exercise recovery response [51, 52]. Treatment of co-cultures with the myogenic inhibitor MSTN results in a pronounced reduction of cell fusion, which is in agreement with previous studies that show a reduction in the myogenic index by treatment with MSTN [21, 53]. Furthermore, cytokine secretion is induced during physiological conditions that are accompanied by myonuclear accretion [52, 54], such as recovery from eccentric exercise and recovery from muscle atrophy. In contrast to the inhibitory effect of the pro-inflammatory cytokine TNFα on myogenesis, the anti-inflammatory cytokines IL-13 and IL-4 have been reported to increase fusion by acting as recruitment factors for muscle growth [29, 30]. In line with this, we find an increase in the relative fusion reporter activity upon co-culture treatment with IL-4 and IL-13. Furthermore, during the recovery phase after simulation of muscle damaging exercise in vitro through electrical pulse stimulation [27], we observe an increase in myonuclear accretion. Moreover, upon induction of in vitro muscle atrophy by treatment with the Pi3K inhibitor LY [55], in line with previous research [56], a reduction in myonuclear accretion in the presence of LY is observed, which may reflect a requirement of IGF-I/AKT signaling in one of the myogenic processes leading to myonuclear accretion. Interestingly, myonuclear accretion is increased during recovery from myotube atrophy upon removal of LY, illustrating that myonuclear accretion can be determined by myotube intrinsic properties, and suggesting a role of myonuclear accretion in restoring myofiber size.

In vivo, contraction-induced muscle damage and muscle atrophy are processes that affect the adult myofiber. However, the subsequent satellite cell response during muscle recovery suggests that crosstalk occurs between muscle fibers and satellite cells. Importantly, the current model system allows the separate treatment of myotubes and myoblasts, and thereby provides the possibility to disentangle cell type-specific signals and responses, and facilitates exploration of their role in the regulation of postnatal myogenesis. We exploited this distinctive capacity by assessing whether Myomaker, a recently identified regulator of fusion, is required for postnatal myonuclear accretion in a cell-type specific manner. To this end, we knocked down Myomaker in myotubes and/or myoblasts before initiation of co-culturing, and report a myoblast specific requirement of Myomaker for postnatal myonuclear accretion. This is in line with an early Myomaker study, which reported a cell-type specific differential requirement of this protein in myoblasts–fibroblasts fusion [32], and a more recent study, which reported a satellite cell-specific requirement for Myomaker in overload-induced muscle hypertrophy [15].

## Conclusions

In summary, we developed a Cre/LoxP-based in vitro model that better reflects postnatal myogenesis by including the relevant fusion partners, and can be used for semi-quantitative assessment of postnatal myonuclear accretion. We show that this model is responsive to known ligands with myogenesis inducing and inhibiting properties, as well as physiological triggers that affect postnatal myonuclear accretion. Moreover, this model allows the distinction the cell type-specific roles of signals and responses in the regulation of postnatal myogenesis. Together, these features make the Cre/LoxP-based in vitro model of postnatal myonuclear accretion suitable for both basal research on the molecular regulation of postnatal myogenesis, as well as translational research on the whole range of muscle diseases that may involve impaired satellite cell number or function.

## Additional files

Additional file 1: Live cell time-lapse imaging. In vitro myoblast–myotube fusion captured by live cell time-lapse imaging. Days and hh:mm indicate time after initiation of myotube-myoblast co-culture. (AVI 213275 kb)

Additional file 2: Figure S1. Selected frames depicting in vitro myoblast–myotube fusion captured by live cell time-lapse imaging. Arrow head indicates myoblast movement, and subsequent fusion with a myotube. T= time (hh:mm) after initiation of myotube-myoblast co-culture. (EPS 14309 kb)

Additional file 3: Figure S2. Hybrid formation in DiD-stained HSM myotubes 2 days after initiation of co-culturing with DiO-stained HSM myoblasts. (DAPI/nuclei: blue; DiD: red; DiO: green). Arrows indicate non-hybrid myotubes, arrow heads indicate hybrid myotubes. (EPS 38624 kb)

Additional file 4: Figure S3. Moving average and variation per well by increasing number of fields of view (FOV). A) moving average of total number of myotubes per well in well 1 (left) and well 2 (right), B) moving average of number of hybrid myotubes per well in well 1 (left) and well 2 (right), C) moving average of % hybrid myotubes per well in well 1 (left) and well 2 (right). (EPS 1757 kb)

Additional file 5: Figure S4. Staining-based assessment of myonuclear accretion 2 days after initiation of co-culturing +/- 10 nM IGF-I treatment started 24 hours before start of co-culturing (T=-24), upon co-culturing (T=0), or 24 hour after start of co-culturing (T=24) (A-C). A) total number of myotubes, B) number of hybrid myotubes, C) % hybrid myotubes. Values are means ± SEM, *n* = 4. *p < 0.05, **p < 0.01, ***p < 0.001 compared to control. (EPS 816 kb)

Additional file 6: Figure S5. Inter-rater reliability of staining-based assessment of postnatal myonuclear accretion. A) scatter plot of total number of myotubes per well by observer 1 and observer 2, B) Bland-Altman plot of total number of myotubes per well, C) scatter plot of number of hybrid myotubes per well by observer 1 and observer 2, D) Bland-Altman plot of number of hybrid myotubes per well, E) scatter plot of % hybrid myotubes per well by observer 1 and observer 2, F) Bland-Altman plot of % hybrid myotubes per well. Means (solid line), and the 95% confidence interval (95% CI) and 95% limits of agreement (95% LOA) (dotted line) are displayed. Inter-rater correlation was tested by Pearson correlation (*R*^2^), absolute inter-rater agreement was assessed by two-way mixed model intraclass correlation coefficient (ICC) with 95% CI, and fixed bias was assessed by Bland-Altman analysis and tested by one sample *t* test. ***p < 0.001. (EPS 1837 kb)

Additional file 7: Figure S6. Optimization of Cre/LoxP-based assessment of myonuclear accretion. Co-culture of LV-floxed-Luc C2C12 myotubes with Cre C2C12 myoblasts (A–D). A) relative luciferase activity (RLU/protein content) per well 2 days after addition of 0, 2500, 5000 or 10000 myoblasts/cm^2^ (*n* = 3). Cells lysed at indicated time points (h) after initiation of co-culturing +/- 10 nM IGF-I (*n* = 8), B) luciferase activity (RLU) per well, C) protein content (μg/μL) per well, D) relative luciferase activity (RLU/protein content) per well. Co-culture of Cre C2C12 myotubes with LV-floxed-Luc C2C12 myoblasts (E-H). E) relative luciferase activity (RLU/protein content) per well 2 days after addition of 0, 2500, 5000, or 10000 myoblasts/cm^2^ (*n* = 3). Cells lysed at indicated time points (hours) after initiation of co-culturing +/- 10 nM IGF-I (*n* = 8), F) luciferase activity (RLU) per well, G) protein content (μg/μL) per well, H) relative luciferase activity (RLU/protein content) per well. Values are means ± SEM. *p < 0.05, **p < 0.01, ***p < 0.001 compared to 0 cells/cm^2^ or compared to *T* = 16 within each condition, and between indicated bars. (EPS 2309 kb)

Additional file 8: Figure S7. Validation of Cre/LoxP-based luciferase production as a reporter of myonuclear accretion. Co-culture of Cre C2C12 myotubes with LV-floxed-Luc C2C12 myoblasts. Cells lysed 3 days after initiation of co-culturing +/- increasing concentrations of TNFα (*n* = 4) (A-C), A) luciferase activity (RLU) per well, B) protein content (μg/μL) per well, C) relative luciferase activity (RLU/protein content) per well. Cells lysed 3 days after initiation of co-culturing +/- increasing concentrations of IGF-I (*n* = 4) (D-F), D) luciferase activity (RLU) per well, E) protein content (μg/μL) per well, F) relative luciferase activity (RLU/protein content) per well. G) myonuclear accretion of BrdU+ myoblast nuclei into myotubes, 3 days after initiation of co-culturing (DAPI/nuclei: blue; BrdU: green). Arrows indicate examples of BrdU+ myoblast nuclei, arrow head indicates a BrdU+ nucleus in a myotube. H) Average number of BrdU+ nuclei in myotubes per well 3 days after initiation of co-culturing +/- 10 nM IGF-I, 50 nM IGF-I, or 100 nM IGF-I. I) scatter plot of the relative luciferase signal (RLU/protein content) and BrdU+ nuclei in myotubes, 3 days after initiation of co-culturing +/- 10 nM IGF-I, 50 nM IGF-I, or 100 nM IGF-I. Abbreviations: r_P_, Pearson correlation coefficient; r_S_, Spearman correlation coefficient. Values are means ± SEM. *p < 0.05, **p < 0.01, ***p < 0.001 compared to control; ^#^*p* < 0.1 for correlations. (EPS 3620 kb)

Additional file 9: Figure S8. Dissection of effects of alterations in fusion, and protein synthesis on the fusion reporter activity. Co-culture of Cre C2C12 myotubes with LV-floxed-Luc C2C12 myoblasts (A-C). Cells lysed at indicated time points (hours) after initiation of co-culturing +/- 10 ng/mL TNFα, 10 nM IGF-I, or 50 nM IGF-I (*n* = 4), A) luciferase activity (RLU) per well, B) protein content (μg/μL) per well, C) relative luciferase activity (RLU/protein content) per well. Cre/LV-floxed-Luc C2C12 myotubes obtained by 5 days co-culturing of Cre C2C12 and LV-floxed-Luc C2C12 myoblasts (D-E). Cells lysed at indicated time points (hours) after start of incubation +/- 10 ng/mL TNFα, 10 nM IGF-I, or 50 nM IGF-I (*n* = 4), D) luciferase activity (RLU) per well, E) protein content (μg/μL) per well, F) relative luciferase activity (RLU/protein content) per well. Values are means ± SEM. Results 2-way ANOVA (treatment × time); A) treatment effect***, time effect***, interaction***, B) treatment effect*, time effect***, interaction^ns^, C) treatment effect***, time effect***, interaction***, D) treatment effect***, time effect***, interaction^ns^, E) treatment effect***, time effect***, interaction*, F) treatment effect***, time effect***, interaction*. Post-hoc analyses with Bonferroni correction are depicted in the figures, *p < 0.05, **p < 0.01, ***p < 0.001 compared to control within each time-point. (EPS 1411 kb)

## Abbreviations

BrdU: Bromodeoxyuridine
BSA: Bovine serum albumin
CytoD: Cytochalasin D
DM: Differentiation medium
DMEM: Dulbecco’s modified Eagle’s medium
EPS: Electrical pulse stimulation
FBS: Fetal bovine serum
FOV: Fields of view
GM: Growth medium
HBSS: Hanks’ Balanced Salt Solution
HSM: Human C25 myoblasts
IGF-I: Insulin-like growth factor 1
IL-13: Interleukin-13
IL-4: Interleukin-4
LY: LY294002
MSTN: Growth differentiation factor 8
PBS: Phosphate buffered saline
PFA: Paraformaldehyde
RT: Room temperature
TBST: Tris-buffered saline
TNF-α: Tumor necrosis factor alpha
